# Environmental Enrichment Effects on Development of Retinal Ganglion Cell Dendritic Stratification Require Retinal BDNF

**DOI:** 10.1371/journal.pone.0000346

**Published:** 2007-04-04

**Authors:** Silvia Landi, Maria Cristina Cenni, Lamberto Maffei, Nicoletta Berardi

**Affiliations:** 1 Scuola Normale Superiore, Pisa, Italy; 2 Istituto di Neuroscienze del Consiglio Nazionale delle Ricerche (CNR), Pisa, Italy; 3 Dipartimento di Psicologia, Università di Firenze, Florence, Italy; University of Washington, United States of America

## Abstract

A well-known developmental event of retinal maturation is the progressive segregation of retinal ganglion cell (RGC) dendrites into a and b sublaminae of the inner plexiform layer (IPL), a morphological rearrangement crucial for the emergence of the ON and OFF pathways. The factors regulating this process are not known, although electrical activity has been demonstrated to play a role. Here we report that Environmental Enrichment (EE) accelerates the developmental segregation of RGC dendrites and prevents the effects exerted on it by dark rearing (DR). Development of RGC stratification was analyzed in a line of transgenic mice expressing plasma-membrane marker green fluorescent protein (GFP) under the control of Thy-1 promoter; we visualized the a and b sublaminae of the IPL by using an antibody selectively directed against a specific marker of cholinergic neurons. EE precociously increases Brain Derived Neurotrophic Factor (BDNF) in the retina, in parallel with the precocious segregation of RGC dendrites; in addition, EE counteracts retinal BDNF reduction in DR retinas and promotes a normal segregation of RGC dendrites. Blocking retinal BDNF by means of antisense oligos blocks EE effects on the maturation of RGC dendritic stratification. Thus, EE affects the development of RGC dendritic segregation and retinal BDNF is required for this effect to take place, suggesting that BDNF could play an important role in the emergence of the ON and OFF pathways.

## Introduction

One of the most remarkable features of visual system parallel processing is the functional segregation of ON and OFF pathways originating in the retina [1]–[3]. This functional segregation has an anatomical correspondence in the stratification of the dendrites of ON- and OFF-center RGCs in different sublaminae of the IPL, sublamina a and b. It is well known that this segregated pattern of arborizations is achieved by a progressive restriction of RGC dendrites from bistratified processes into monostratified arborizations during retinal development [4].

The factors regulating this striking morphological rearrangement are not known, although glutamatergic [3], [5] and cholinergic transmission [6] and visually driven activity [7] have been demonstrated to play a role.

Our recent studies [8] have shown that an increased stimulation, such as that provided by EE, can affect the development of retinal visual responses. Whether RGC dendritic segregation is sensitive to the experience provided by EE is not known. Landi et al. [8] also found that EE precociously increases BDNF expression in the retina and that these higher levels of BDNF are crucial for triggering retinal functional development, suggesting an involvement of retinal BDNF in the experience-dependent maturation of retinal circuitry.

In the present study we investigated whether the developmental remodelling of RGC dendrites is sensitive to the experience provided by EE and whether BDNF is involved in mediating EE effects.

We found that EE accelerates the developmental segregation of RGC dendritic arborizations and counteracts the blockade of this process induced by DR. EE precociously increases BDNF in the retina, in parallel with the precocious segregation of RGC dendrites; moreover, EE counteracts retinal BDNF reduction caused by DR. Blocking retinal BDNF expression by means of antisense oligonucleotide injections in EE animals prevents EE from accelerating RGC dendritic developmental segregation.

These results show that the developmental transition of RGC dendrites from the initial bistratified to the final monostratified pattern is sensitive to environmental experience, and identifies retinal BDNF as a key factor mediating experience effects on RGC maturation.

## Results

### Segregation of RGC dendritic stratification

**Figure 1:**
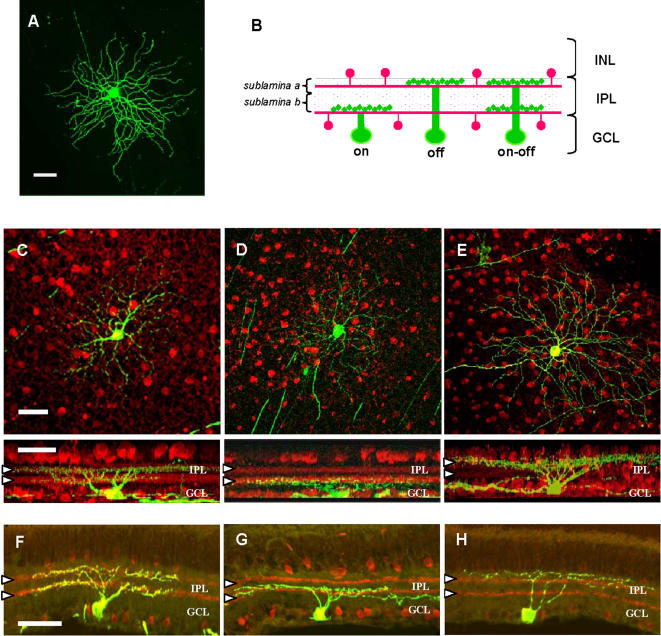
Dendritic stratification pattern of RGCs in Thy-1 mGFP mice. (A) Confocal image of an RGC from whole-mount retina of a mGFP mouse. GFP expression is enhanced with a specific immunostaining. Immunostaining with anti-GFP antibody shows that RGC somata, dendrites and axons are GFP-positive [scale bar = 50 µm]. (B) Schematic representation of the patterning of cholinergic amacrine cell projections (red), which identify the a and b sublaminae of the IPL. An ON- and an OFF-center RGC, with dendrites monostratified in the b or a sublaminae, respectively, and an ON-OFF bistratified RGC are drawn in green. GCL: ganglion cell layer; IPL: inner plexiform layer; INL: inner nuclear layer. (C, D, E): Top row: examples of RGC (green) confocal reconstructed maximum projection images taken from whole-mount retinas from P16 mGFP mice; bottom row: 90 degrees rotation images of the cells displayed above. Red label denotes the immunolabeling pattern of Choline Acetyltransferase (ChAT) positive amacrine cells. Confocal microscopy was used to produce stacked images of three-dimensional reconstructed GFP-expressing RGCs and of ChAT positive amacrine cells. ChAT positive cell bodies are respectively in the GCL and in the INL, while their projections form two bands clearly visible in the rotated images (white arrow heads) that run along the sublamina a and b of the IPL. Bistratified RGCs present a double-layered segregated arborization with respect to the two anti-ChAT labeled bands (C, bottom), while monostratified ganglion cells have their dendrites proximal to the cell body and restricted to the ChAT positive band within sublamina b (D, bottom) or distal to the cell body and restricted to the outermost ChAT positive band in sublamina a (E, bottom) [scale bars = 50 µm]. (F, G, H) Examples of RGC (green) confocal images taken from 25 µm vertical retinal sections from P30 mGFP mice. The red bands representing the projections of cholinergic amacrine cells immunolabeled with ChAT, which denote the sublaminae of the IPL, are pointed at with white arrow heads [scale bar = 50 µm].

We analysed the segregation of RGC dendrites in bistratified and monostratified processes into a and b sublaminae of the IPL at different ages after birth. We used a transgenic line of mice expressing plasma-membrane marker green fluorescent protein (mGFP) under control of Thy-1 promoter [9]. GFP consistently labels dendrites, somata and axons of the RGCs, as shown in Fig. 1A.

We visualized and quantified the stratification pattern of RGCs in retinal vertical sections (Fig. 1F, 1G, 1H) for all ages and also in whole-mount retinas at P16 (Fig. 1C, 1D, 1E); for each retina all GFP-positive cells were analysed with the exception of the extremely rare adjacent cells. In both procedures (vertical sections and whole-mount), the a and b sublaminae of the IPL were identified by using an antibody selectively directed against a specific marker of cholinergic neurons, the choline acetyl-transferase (ChAT; red labelling in Fig. 1); indeed, the projections of cholinergic amacrine cells run along the two sublaminae inside the IPL [1], [2], [10], [11], [6] and are present very early in retinal development [12], [11]. We have used the two reference planes provided by ChAT immunostained projections to detect RGCs as bistratified or monostratified. While bistratified RGCs present a double-layered segregated arborisation with respect to the two anti-ChAT labelled bands (Fig. 1C and F), monostratified RGCs have dendrites restricted to the ChAT positive band inside sublamina b (Fig. 1D and G) or around the outermost ChAT positive band in sublamina a (Fig. 1E and H).

### RGC dendritic stratification develops postnatally

**Figure 2:**
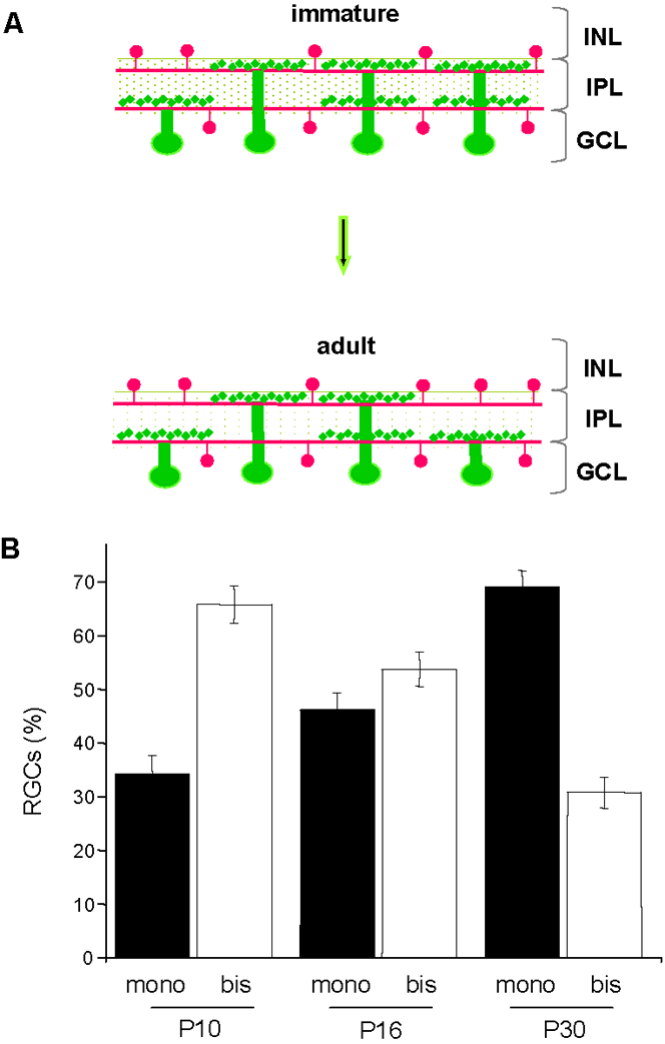
RGC stratification during postnatal development in normal non-EE Thy-1 mGFPmice. (A) Schematic representation illustrating the passage from immature to adult state during development of RGC dendritic stratification (cholinergic amacrine cells in red, RGCs in green). (B) Percentages of monostratified and bistratified RGCs during development in normal non-EE mice between P10 and P30 are respectively 34,2±3,5% at P10 (N = 4), 46,2±3,2% at P16 (N = 4), 69,2±2,9% at P30 (N = 5) for monostratified cells, and 65,8±3,5% at P10, 53,8±3,2% at P16, 30,8±2,9% at P30 for bistratified cells. Vertical bars indicate SEM. There is a significant decline of bistratified RGCs with age (One Way ANOVA, p<0,001). The size of the RGC sample (total number of RGCs analyzed) and the percentage of bistratified RGCs are reported for each retina in Table 1.

An age-dependent decline of bistratified RGCs has been observed in cat [13], [5], ferret [14], primate [15] and mouse [7], as is schematically illustrated in Fig. 2A. In particular, Tian and Copenhagen [7] found a decrease of bistratified RGCs from more than 50% at P10 to 29% at P30.

We observed a comparable developmental decline of bistratified RGCs in the mGFP mice reared in standard conditions (non-EE mice). Our analysis revealed that in P10 mice 65,8% of RGCs were bistratified, while at P16 and at P30 this percentage was 53,8% and 30,8%, respectively, as indicated in Fig. 2B (One Way ANOVA, p<0,001). The total number of RGCs analyzed and the percentage of bistratified RGCs are reported, for each retina, in Table 1.

**Table 1:** Percentage of bistratified RGCs during development in non-EE and EE mice.

non-EE P10	non-EE P16		non-EE P30
1) 69,4% (34/49)	1) 55,8% (43/77)	**1wm) 50% (12/24)**	1) 32% (16/50)
2) 73,2% (30/41)	2) 50% (26/52)	**2wm) 53,1% (17/32)**	2) 40,9% (18/44)
3) 57,1% (8/14)	3) 47,4% (9/19)	**3wm) 51,4% (19/37)**	3) 28% (7/25)
4) 63,6% (7/11)	4) 61,9% (13/21)		4) 23,3% (7/30)
			5) 30% (6/20)
*Average*±*SEM*	*Average*±*SEM*	***Average*** **±** ***SEM (wm)***	*Average*±*SEM*
65,8%±3,5	53,8%±3,2%	**51,5%±0,9%**	30,8%±2,9
*Total cells*	*Total cells*	***Total cells (wm)***	*Total cells*
79/115 (68,7%)	91/169 (53,8%)	**48/93 (51,6%)**	54/169 (32%)
**EE P10**	**EE P16**		**EE P30**
1) 50% (5/10)	1) 56% (14/25)	**1wm) 42,5% (17/40)**	1) 27,4% (17/62)
2) 52,4% (11/21)	2) 28,3% (15/53)	**2wm) 36,8% (7/19)**	2) 33,3% (13/39)
3) 45,5% (10/22)	3) 30,4% (17/56)	**3wm) 34,2% (13/38)**	3) 37,8% (14/37)
4) 42,1% (16/38)	4) 25,8% (8/31)		
5) 31,3% (5/16)	5) 42,9% (12/28)		
*Average*±*SEM*	*Average*±*SEM*	***Average*** **±** ***SEM (wm)***	*Average*±*SEM*
*44,2%*±3,7%	36,7%±5,7%	**37,8%±4,2%**	32,9%±3
*Total cells*	*Total cells*	***Total cells (wm)***	*Total cells*
47/107 (43,9%)	66/193 (34,2%)	**37/97 (38,1%)**	44/138 (31,9%)

Data in bold are the percentage of bistratified RGCs obtained from whole-mount retinas (wm). For each retina, the total number of RGCs analyzed and the percentage of bistratified RGCs are indicated; for each group, the mean percentage of bistratified RGCs and the total number of RGCs analyzed are also reported.

### EE counteracts DR effects promoting RGC dendritic maturation

Recent studies show that DR blocks the developmental remodelling of RGC dendritic stratification: P30 dark reared mice have a percentage of bistratified RGCs not different from that of P10 ones [7]. Since EE prevents DR effects on visual cortical maturation [16], we first examined whether DR effects on RGC stratification could be counteracted by EE.

**Figure 3:**
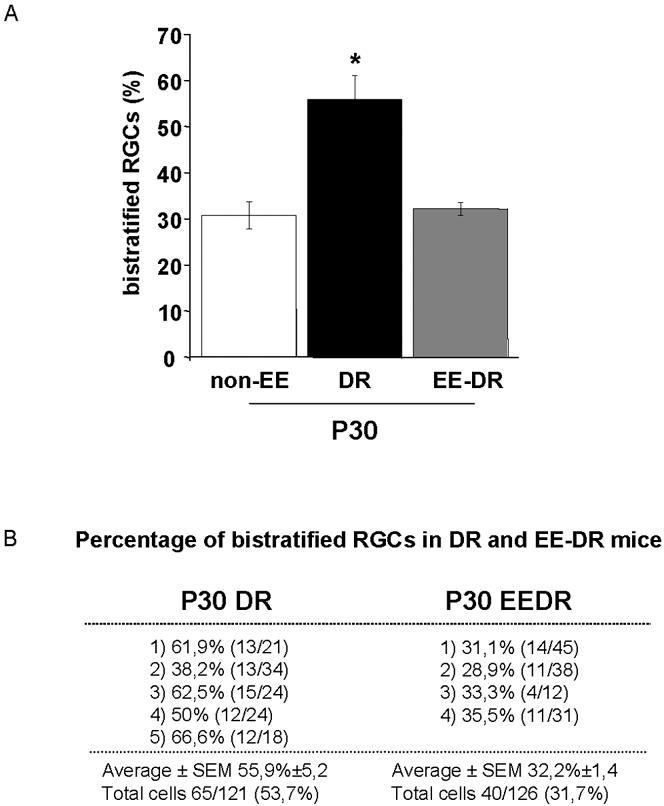
EE counteracts DR effects promoting RGC dendritic maturation. (A) The average percentage of bistratified RGCs in normal non-EE (white), DR (black), and EE-DR mice (grey) at P30. The percentage of bistratified RGCs is 30,8±2,9% in non-EE mice (N = 4, data replotted from Fig. 2); DR blocks RGC dendritic stratification (bistratified cells 55,9±5,2% at P30, N = 5 mice, 65/121 cells), while this process takes place normally in EE-DR mice (P30 EE-DR mice: bistratified cells 32,2±1,4%, N = 4, 40/126 cells). One Way ANOVA shows a statistically significant difference between normal non-EE and DR, and between EE-DR and DR mice; EE-DR are not different from non-EE mice (One Way ANOVA, p<0,001; *post-hoc* Tukey's test). The bars indicate SEM. EE from birth prevents DR effects on the developmental remodelling of RGC dendrites. (B) Percentage of bistratified RGCs and sample size for each retina in DR and EE-DR mice. Data for non-EE mice are in Table 1.

Non-EE or EE mice were dark reared from birth (DR mice or EE-DR mice, respectively) and the percentage of bistratified RGCs was analysed at P30. Control mice of the same age reared in standard conditions under a normal light-dark cycle (normal mice, non-EE) were also analyzed. The size of the RGC sample (total number of RGCs analyzed), the number of RGCs analyzed for each retina and the percentage of bistratified RGCs (per retina and total) are reported in Fig. 3B for DR and EE-DR mice. As expected, DR delays the developmental decrease in the percentage of bistratified RGCs (55,9% in DR mice *versus* 30,8% in normal mice, Fig. 3A); however, EE-DR animals have a percentage of bistratified RGCs which is not different from that of normal mice of the same age (32,2% in EE-DR *versus* 30,8% in normal mice; One Way ANOVA, p<0,001; *post-hoc* Tukey's test).

These results demonstrate that EE prevents DR effects on the developmental remodelling of RGC dendrites.

### EE affects the maturational refinement of RGC dendrites

**Figure 4:**
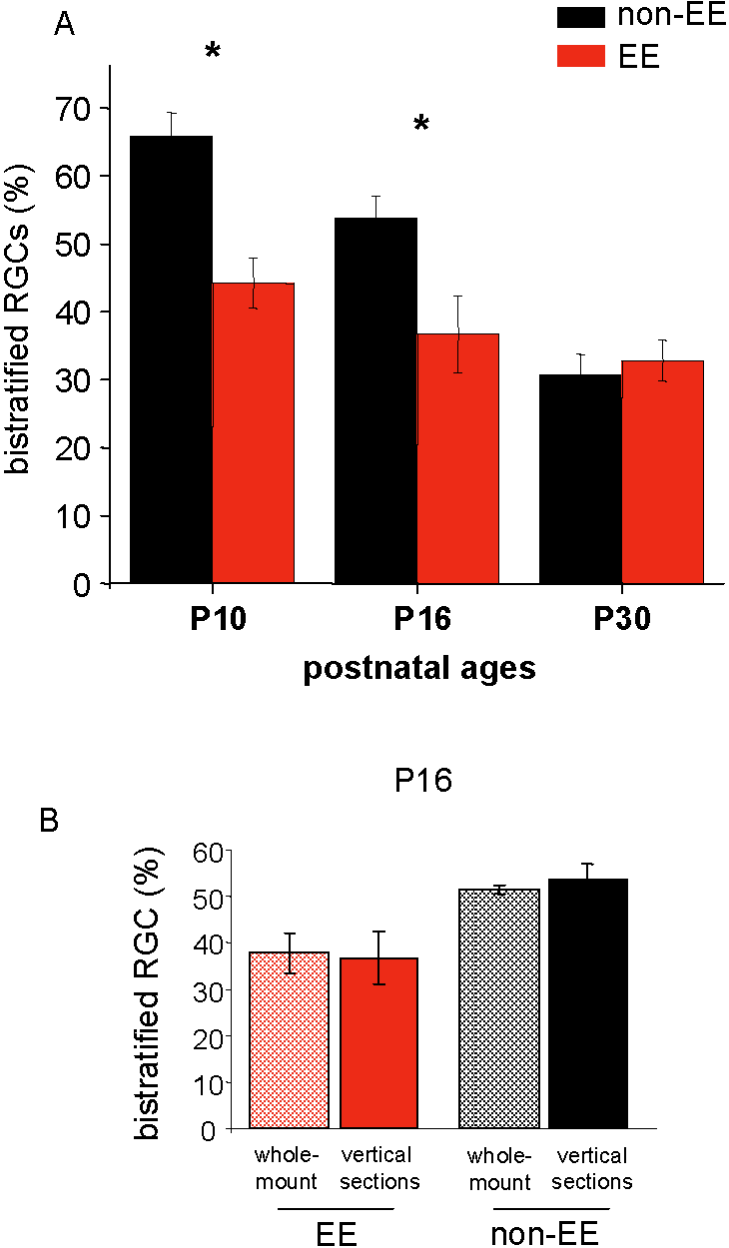
EE affects the maturational refinement of RGC dendrites. (A) Mean percentage of bistratified RGCs in non-EE (black) and EE mice (red) at P10 (non-EE: 65,8±3,5%, N = 4, 79/115 cells; EE: 44,2±3,7%, N = 5, 47/107 cells), P16 (non-EE: 53,8±3,2%, N = 4, 91/169 cells; EE: 36,7±5,7%, N = 5, 66/193 cells) and P30 (non-EE: 30,8±2,9%, N = 5, 54/169 cells; EE: 32,9±3%, N = 3, 44/138 cells). Two Way ANOVA shows a significant effect of age (p = 0,006) and environmental housing condition (p<0,001). *Post-hoc* Tukey's test reveals a significant difference between EE and non-EE at P10 and P16 (asterisk). The bars indicate SEM. EE accelerates the process of the segregation of RGC arborizations. (B) Mean percentage of bistratified RGCs in non-EE (hatched, black, N = 3, 51,5±0,9%, 48/93 cells) and EE (hatched, red, N = 3, 37,8±4,2%, 37/97 cells) P16 mice obtained from confocal reconstructed images in whole-mount retinas after digital rotation, as exemplified in Fig. 1. Data obtained in retinal vertical sections are replotted from A for direct comparison (solid bars). There is no difference between the results obtained with these two methods of dendritic stratification analysis (Two Way ANOVA, housing×method, housing p = 0,006, method p = 0,911; no significant interaction). The size of the RGC sample and the percentage of bistratified RGCs are reported, for each retina, in Table 1.

To better understand the influence of EE on RGC circuitry development, we analyzed the maturation of RGC dendritic stratification in EE and non-EE mice. We found that the process of RGC dendritic stratification occurs much earlier in EE than in non-EE mice. In P10 EE mice the percentage of bistratified RGCs is much lower than in P10 non-EE mice (44,2% and 65,8%, respectively, Fig. 4A and TABLE 1) and the incidence of bistratified RGCs in P16 EE mice does not differ from that of P30 non-EE mice (36,7% *versus* 30,8%, Fig. 4A) or from that of P30 EE mice (32,9%), indicating that by P16 the developmental segregation of RGC dendritic stratification is already completed in EE mice (Two Way ANOVA, housing×age, effect of age, p = 0,006, effect of housing, p<0,001; no interaction; *post-hoc* Tukey's test). The accelerated completion of RGC dendritic stratification at P16 in EE mice was confirmed in retinas analyzed with a different procedure, analyzing RGC dendritic stratification through the entire thickness of the GCL and IPL by confocal microscope in whole-mount ChAT immunostained retinas. We found that the percentages of bistratified RGCs in whole-mount retinas and in retinal vertical sections did not significantly differ, both in non-EE and EE P16 mice, as reported in Fig. 4B and Table 1 (Two Way ANOVA, housing per method; housing, p = 0,006, method p = 0,911; no interaction; *post-hoc* Tukey's test).

It is important to note that the final value of bistratified RGCs at P30 does not differ between EE and non-EE mice, suggesting that EE affects the time course of the segregation process and not its final outcome.

We did not observe any differences in the thickness of the IPL between EE and non-EE mice, estimated in retinal vertical sections (at P10, EE: 61,8 µm±1,5; non-EE: 65,7±2,4; at P16, EE: 62,6±2,2; non-EE: 59,7±1,8; Two Way ANOVA, housing per age, p = 0,612). These data indicate that the changes in RGC dendritic segregation induced by EE reflect a true variation in RGC circuitry rather than alterations in retinal architecture.

Thus, EE influences the maturational remodelling of RGC dendrites by accelerating the segregation of RGC arborizations.

### Retinal BDNF is required for the RGC circuitry maturation promoted by environmental experience

BDNF is an important factor in neuronal dendritic development [17]–[20] and data from the Xenopus have shown that retinal BDNF reduces RGC dendritic arborization [21], [22]. Interestingly, our recent study showed that BDNF expression in the retina is increased by EE in developing animals and these higher levels of BDNF are crucial for triggering retinal functional development [8].

We therefore asked whether BDNF could be involved in the effects produced by EE on the developmental stratification of RGC dendrites.

We first analysed whether EE effects on RGC dendritic maturation in DR mice were correlated with changes in BDNF expression in the RGC layer. Previous findings had already shown that DR modulates retinal BDNF expression, decreasing its levels in the rat retina [23]. We tested whether EE, which promotes a normal RGC dendritic segregation in DR animals, counteracting DR effects on RGC circuitry development, counteracted also DR effects on BDNF expression.

**Figure 5:**
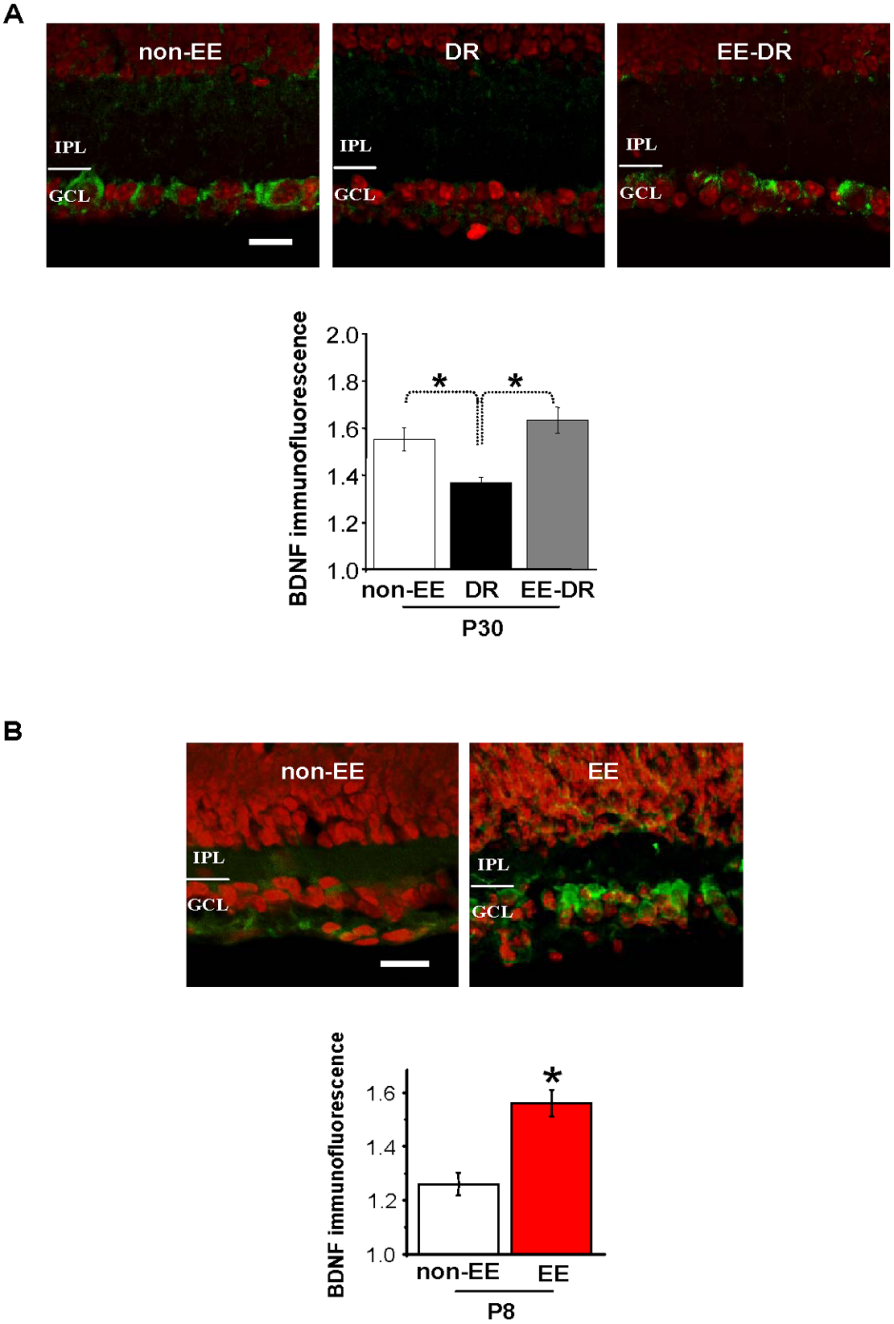
BDNF expression is regulated by EE. (A) *Top*: Vertical sections through the retina of P30 DR mice are immunostained for BDNF (green) and labelled with a nuclear marker (TOTO, red). BDNF immunoreactivity in RGC layer is remarkably lower in DR (center) than in normal non-EE mice (left), while RGC layer of EE-DR mice (right) shows a level of BDNF immunofluorescence similar to that of normal mice. Scale bar: 20 µm. *Bottom:* Quantitative analysis of BDNF immunofluorescence intensity normalized to background level in the RGC layer of normal non-EE (white), DR (black) and EE-DR mice (grey). BDNF immunofluorescence in the RGC layer of DR mice is significantly lower than in non-EE and EE-DR mice; no difference was found between non-EE and EE-DR mice (ANOVA on Ranks, p<0,001; Dunn's Method). (B) *Top*: Vertical sections through the retina of P8 mice. Sections are immunostained for BDNF (green) and labelled with a nuclear marker (TOTO, red). BDNF immunoreactivity is lower in the RGC layer of non-EE mice (top) with respect to that in the RGC layer of EE mice of the same age. Scale bar: 20 µm. *Bottom*: Quantitative analysis of BDNF immunofluorescence intensity normalized to background level in the RGC layer of non-EE (white) and EE mice (red). t-test shows a statistical difference (asterisk) between the two groups (p<0.001). The bars indicate SEM.

Non-EE or EE mice were dark reared from birth and the expression of BDNF in the retina was analysed by immunohistochemistry at P30. BDNF expression in the retina of P30 mice normally reared in a light-dark cycle (control mice, non-EE) was also assessed for comparison. As expected, DR reduces BDNF protein expression in the retina with respect to controls: BDNF immunoreactivity in the RGC layer was statistically different between P30 DR and control mice (respectively, 1,37±0,02 *versus* 1,55±0,05); however, BDNF expression in EE-DR animals did not differ from that in control mice at the same age (1,63±0,05) (ANOVA on Ranks, p<0,001; Dunn's Method; see Fig. 5A) indicating that BDNF reduction in DR mice is completely counteracted by EE.

We then analyzed whether the accelerated segregation of RGC dendrites in EE mice was preceded by an enhanced expression of BDNF in the RGC layer. Since EE effects on RGC segregation are already evident at P10, we examined BDNF expression at an earlier age, P8. We found higher levels of BDNF protein in the retina of EE mice compared with those of non-EE mice (1,56±0,05 *versus* 1,26±0,04; t-test, p<0,001), as shown in Fig. 5B.

**Figure 6:**
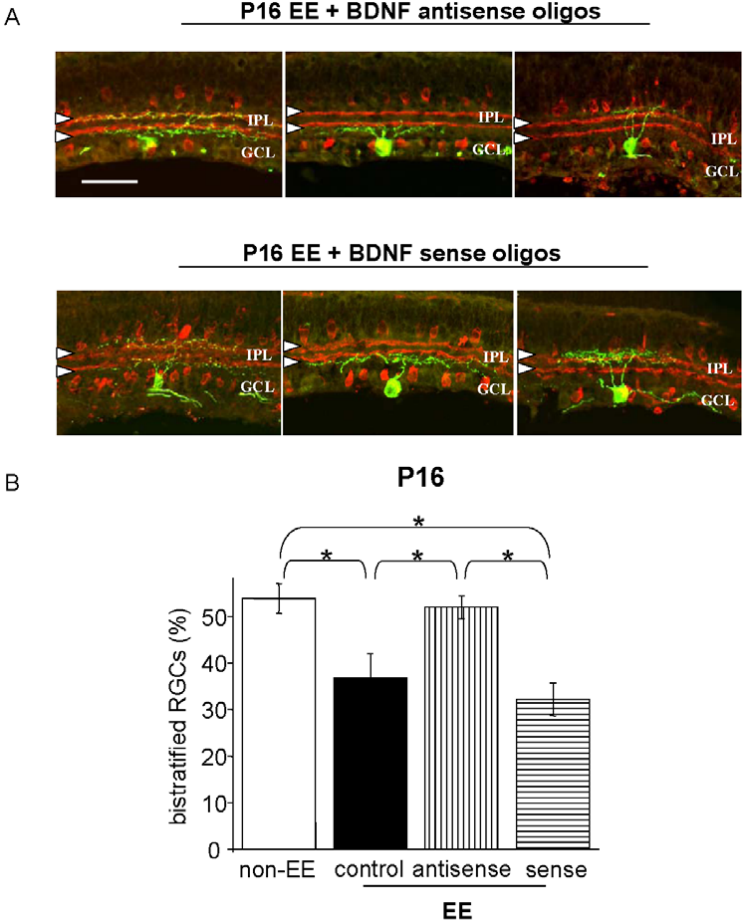
BDNF mediates the effects of EE on RGC dendritic segregation. (A) Examples of an ON-OFF RGC (left), an ON RGC (center), an OFF RGC (right) in retinal vertical sections of P16 EE antisense treated mice (top row) or sense treated mice (bottom row) (scale bar: 50 µm). Conventions as for Fig. 1. (B) Average percentage of bistratified RGCs in P16 non-EE mice (white), untreated EE mice (control, black), EE mice treated with BDNF antisense (antisense, vertical line-pattern) and EE mice treated with BDNF sense (sense, horizontal line-pattern). In the retinas of EE mice injected with BDNF antisense oligos (N = 5) the percentage of bistratified RGCs is similar to that of normal non-EE mice of the same age (51,9±2,5%, 43/84 cells *versus* 53,8±3,2%, 91/169 cells), whereas the control treatment with sense oligos (N = 5) has no effect on the accelerated development produced by EE (32,2±3,5%, 29/88 cells *versus* 36,9±5,2%, 31/81 cells in EE mice treated with BDNF sense oligos and EE untreated mice, respectively). One Way ANOVA indicates a statistical difference (asterisks) between control EE and antisense treated EE, between sense and antisense treated EE mice and between non-EE and control or sense treated EE mice; no difference is found between untreated (control) and sense treated EE mice and between non-EE and antisense treated EE mice (p = 0,001; *post-hoc* Tukey's test). The bars indicate SEM. The blockade of BDNF expression blocks the effects of EE on RGC dendritic stratification.

To assess whether this BDNF increment was necessary for the effects of EE on the development of RGC dendritic arborization to take place, we decreased BDNF expression in the retina of EE mice by injecting antisense oligonucleotides against BDNF, with the same protocol previously employed to suppress BDNF protein levels in the developing rat retina [24], [25], [8] and in particular, to block EE effects on the development of retinal acuity [8]. Antisense oligonucleotides against BDNF were injected intraocularly from P6 to P12, and the percentage of bistratified RGCs was assessed at P16. Injections of sense oligonucleotides were used as controls (Fig. 6A).

We found that BDNF antisense oligos blocked the accelerated stratification observed in EE retinas: the percentage of bistratified cells in P16 EE mice treated with BDNF antisense oligos (51,9%) was significantly higher than in untreated EE mice (36,8%), while it did not differ from that in P16 non-EE mice (53,8%) (t-test, p = 0,648). By contrast, the percentage of bistratified RGCs (32,2%) in P16 EE mice treated with BDNF sense was not statistically different from that of P16 EE untreated mice and significantly lower than in P16 EE mice treated with BDNF antisense (One Way ANOVA, p = 0,001; *post-hoc* Tukey's test) (Fig. 6B and Table 2).

**Table 2:** Percentage of bistratified RGCs in EE BDNF antisense and sense-treated mice

Age P16		
EE untreated (control)	EE BDNF antisense-treated mice	EE BDNF sense-treated mice
1) 28,6% (4/14)	1) 52,6% (10/19)	1) 26,6% (4/15)
2) 47,3% (9/19)	2) 56,2% (9/16)	2) 22,2% (4/18)
3) 50% (9/18)	3) 43,5% (10/23)	3) 40,9% (9/22)
4) 23% (3/13)	4) 50% (6/12)	4) 38,1% (8/21)
5) 35,3% (6/17)	5) 57,1% (8/14)	5) 33,3% (4/12)
*Average*±*SEM*	*Average*±*SEM*	*Average*±*SEM*
36,9±5,2%	51,9±2,5%	32,2±3,5%
*Total cells*	*Total cells*	*Total cells*
31/81 (38,3%)	43/84 (51,2%)	29/88 (32,9%)

Percentage of bistratified RGCs in P16 EE mice treated with BDNF antisense oligos, with BDNF sense oligos or untreated (EE). For each retina, the total number of RGCs analyzed and the percentage of bistratified RGCs are indicated; for each group, the mean percentage of bistratified RGCs and the total number of RGCs analyzed are also reported.

Thus, these results suggest that the effects of EE on RGC dendritic developmental stratification are dependent on retinal BDNF.

## Discussion

Retinal development has long been considered to be insensitive to experience. Recent evidences challenging this notion have come from works showing that DR can influence refinement of ON and OFF pathways in the retina [7] and that an increased stimulation, such as that provided by EE, can affect the development of retinal visual responses [8].

In the present work we show that EE accelerates the developmental segregation of RGC dendrites and prevents the blockade of this maturational remodelling caused by DR.

Our results, as well as confirming the susceptibility of the developing retina to experience, increase our knowledge on EE effects on the development of sensory systems and show for the first time that a manipulation as simple as enriching the environment affects the development of RGC dendritic segregation, cardinal feature of retinal circuitry development.

The effects of EE on RGC dendritic stratification are evident already at P10, that is, before eye opening. This precocious action of EE on retinal development is in accordance with our recent results [8] showing that exposure to EE up to P10 is sufficient to trigger the acceleration of retinal visual responses.

This, together with the result that EE leads to normal RGC dendritic stratification in DR mice, suggests that the factors EE acts upon to trigger the refinement of RGC circuitry do not require visual experience, although visual experience may modulate their expression.

Our results identify one of these factors as retinal BDNF. We present three evidences in favour of BDNF being a key factor mediating EE effects on the morphological remodelling of RGC dendritic arborizations, two correlative and one causal. BDNF is precociously increased in the retina of EE mice, as recently found for EE rats [8], in parallel with the precocious segregation of RGC dendrites promoted by EE; BDNF expression, which is reduced in DR retinas [23, our present results], is normal in the retinas of dark reared EE animals, in parallel with the normal segregation of RGC dendrites promoted by EE; the causal evidence is that blocking retinal BDNF expression by means of antisense oligonucleotides prevents EE from accelerating this developmental segregation.

Thus, BDNF increase in EE retinas seems necessary for the effects of EE on RGC dendritic development to take place, indicating that BDNF plays an important role in regulating the developmental transition of RGC dendrites from bi- to mono-stratified.

It is important to underline that the expression of BDNF is affected by EE within the first days of postnatal life when pups are still immobile and dependent on the mother. Indeed, we have found that BDNF protein level is enhanced very precociously (around P8) in the retina of enriched mice. This result is in agreement with our previous study indicating that BDNF protein level is enhanced at very early age by EE: at P7 in the visual cortex of enriched mice [26] and at P10 in the retina of enriched rats [8]. A possible explanation for the precocious enhancement of BDNF is suggested by the combination of two sets of results; we have recently shown that EE animals are subjected to higher levels of maternal care [27], while Liu et al. [28], [29] have shown that high levels of maternal care enhance BDNF mRNA expression in rat hippocampus.

How could changes in BDNF regulate RGC structural refinement?

BDNF could act directly on RGCs in an autocrine fashion. It is known that mRNA for BDNF and its functional receptor, TrkB, are present in RGCs [30], [31] and that RGCs are sensitive to BDNF. In particular, BDNF shapes dendritic morphology [17]–[20] and data from the Xenopus have shown that retinal BDNF reduces RGC dendritic arborization [21].

An interesting possibility is that BDNF control on RGC dendritic developmental remodelling takes place through an action on the synaptic transmission between bipolar cells and RGCs. Afferent input of bipolar cells has been demonstrated to play a critical role in the developmental segregation of RGC dendrites. The bipolar terminals are stratified even before ribbon synapses appear in the IPL [32], [33] and even in the absence of RGCs [33] suggesting that bipolar cells stratify their axon terminals in the IPL responding to molecular cues. The stratification process of RGC dendrites starts at a time when bipolar cells form the first synaptic contacts with RGCs. In addition, injections of APB, a group III metabotropic glutamate receptor agonist, that hyperpolarizes both ON cone and rod bipolar cells preventing their release of glutamate, blocks RGC dendritic stratification [3], [5]. Although specific TrkB labelling was not reported for bipolar cells, making an action of retinal BDNF on these cells unlikely, BDNF might play a role in the development of synaptic transmission between bipolar and RGCs promoting the activation of functional NMDA receptors in RGCs [34].

In addition to the glutamatergic transmission between bipolar and RGCs, also cholinergic transmission, originated by the cholinergic starburst amacrine cells, may play a role in the developmental remodelling of RGC dendritic stratification. The ON-OFF stratification in RGCs is altered in mice lacking the β2 nicotinic receptor subunit [6]. ChAT positive processes stratify into two separate bands, corresponding to sublaminae a and b in the IPL, very early in the developing retina [6], [35], [36], suggesting that starburst neurites could provide local cues for RGC dendrites in the IPL. Recently, it has also been shown that a moderate rearranging of the spatial organization of the two cholinergic bands in the IPL occurs following visual deprivation [37], a manipulation which delays the maturation of RGC dendritic stratification [7]. However, cell specific TrkB labelling was not reported for cholinergic amacrine cells and the morphology of the plexuses of cholinergic projections is unchanged by exogenous BDNF delivered from P8 to P14 [38].

Thus, a direct action of BDNF onto cholinergic amacrine cells seems unlikely; however an indirect action mediated by dopaminergic amacrine cells is possible.

Dopaminergic amacrine cells express TrkB receptor and BDNF clearly controls the development of the retinal dopaminergic network [39]. The projections of dopaminergic cells have been shown to innervate the IPL sublaminae with a temporal order overlapping the time period during which glutamatergic and cholinergic systems begin to mature and RGC dendrites segregate [39], [40]. Dopaminergic amacrine cells have neuromodulatory effects on RGCs via amacrine intermediaries, particularly the AII cells [41], [40]. Recently, an action of dopamine on the acetylcholine release in the retina has been reported [40], suggesting that the retinal dopaminergic tone could affect the cholinergic transmission, and thus influence RGC dendritic stratification.

## Materials and Methods

### Animal handling and treatments

All experiments were performed on mice in accordance with the Italian Ministry of Public Health guidelines for care and use of laboratory animals. Mice lived in an animal house with a temperature of 21°C, 12/12 light/dark cycle and food and water available ad libitum.

We have used line 21 of the transgenic mice expressing plasma-membrane marker green fluorescent protein under control of Thy-1 promoter [Thy-1-mGFP^single^ mice, kindly provided by P. Caroni, Friedrich Miescher Institute, Basel, Switzerland]. GFP is expressed in RGCs in this transgenic line.

Female mice were put with males (one male for every mating cage) in standard cages for reproduction (26×42×18 cm). Pregnant mothers were assigned to four different rearing conditions:

1. standard condition (non-EE): dams with their offspring are housed in standard laboratory cages.
2. enriched condition (EE): 7 days before estimated delivery, pregnant females are transferred to an enriched cage, a large mesh cage (44×62×28 cm) containing several food hoppers, a running wheel and differently shaped objects (tunnels, shelters, stairs) that are completely substituted once a week. At least two-three pregnant mothers are housed into the enriched cage, together with four-five filler females.
3. dark rearing condition (DR): pregnant females are transferred to a standard cage in a dark climatized lightproof environment 7 days before estimated delivery and litters are dark-reared until postnatal day 30 (P30). All manipulations are done with infrared viewers.
4. dark rearing in enriched condition (EE-DR): pregnant females are transferred to an enriched cage in a dark climatized lightproof environment 7 days before estimated delivery; litters are dark reared until P30.

We have used a total of 79 mice; for each animal, one retina was analyzed.

### Immunohistochemistry

Mice were anaesthetized with chloral hydrate (0.2 ml/10g) and perfused transcardially with PBS followed by fixative containing 4% paraformaldehyde in 0.1 M phosphate buffer (pH = 7.4). Eyes were gently removed.

### GFP and ChAT immunohistochemistry

For the analysis of RGC dendritic stratification, GFP and ChAT immunostaining was performed both on retinal cross (vertical) sections and whole-mount retinas. For vertical sections, eyes were postfixed in 4% paraformaldehyde (24 h) and cryoprotected in 30% sucrose. Serial vertical sections 25 µm thick were cut with a cryostat. After a blocking step with 0.3% Triton X-100, retinal sections were incubated overnight at 4°C with goat anti-choline acetyltransferase (ChAT) polyclonal antibody (1∶200, Chemicon) and with rabbit anti-GFP polyclonal antibody (1∶500, Molecular Probes). The first antibody was detected by incubating sections with Alexa Fluor 568 donkey anti-goat IgG (1∶400, Molecular Probes), while the second one was revealed with biotinylated donkey anti-rabbit (1∶200, Vector Lab) followed by extravidin-FITC (1∶300, Sigma Aldrich). For whole-mount preparations, eyes were dissected and the retina isolated and processed as indicated above, using longer incubation periods (3–4 days).

### BDNF immunohistochemistry

Retinal BDNF expression was assessed at P8 in 5 normal non-EE mice and 4 EE mice and at P30 in 4 normal non-EE mice, 6 DR mice and 4 EE-DR mice.

After a blocking step, sections were incubated overnight in chicken polyclonal anti-BDNF antibody (1∶400, Promega), then exposed to the biotinylated donkey anti-chicken IgG (1∶200, Promega) followed by extravidin FITC (1∶300, Sigma Aldrich). Selected BDNF-immunolabelled sections were counterstained with a nuclear marker (TOTO-3, Molecular Probes) to label cell somata. Immunostaining was performed for EE and control non-EE retinal vertical sections in parallel.

### Intraocular injections of oligonucleotides

To study BDNF role in the maturation of RGC dendritic segregation promoted by EE, EE mice received intraocular injections of BDNF antisense or sense phosphorothioate oligonucleotides (Eurogentec, 500 µM; injection volume: at P6, 250 nl, at P9, 500 nl and at P12, 750 nl according the size of the eye camera; estimated oligo concentration in the eye: 25 µM; [24]).

Antisense oligonucleotides have been previously used in various tissues, including retinal tissue, as therapeutic agents, and as research tools for downregulating certain genes [42]–[45]. Antisense oligonucleotides can be successfully introduced into the retina intravitreally [42], [46], [44]; they appear to penetrate in the entire thickness of the retina and in particular in RGC layer and are found intracellularly in RGCs [46], [44]. Moreover, recent findings from our laboratory [24], [25], [8] have demonstrated that intraocular injection of BDNF antisense oligos reduces retinal expression of BDNF in rats during postnatal development.

Injections were performed under ether anaesthesia at P6, P9 and P12. Intraocular injections of BDNF sense and antisense oligos were performed using a glass micropipette inserted at the ora serrata connected to a Hamilton syringe. Sequences of the BDNF antisense -to reduce BDNF mRNA translation- and sense oligonucleotides (targeted to the BDNF translation initiation codon) were 5′-CATCACTCTTCTCACCTGGTGGAAC-3′ and 5′-GTTCCACCAGGTGAGAAGAGTGATG-3′, which correspond to nucleotides 51–75 of the BDNF mRNA and are the same previously used to effectively reduce retinal BDNF levels [24], [25], [8]. Fully phosphorothioate oligonucleotides were dissolved in saline with stock solutions of 1mM. Stock solutions were preserved at −80°C and diluted in saline at the desired concentration at the time of the injection. Analysis of RGC stratification was made at P16 when the accelerated developmental stratification promoted by EE seems completed (see Fig. 4A).

### Analysis of RGC dendritic arborizations

In the GFP transgenic mice employed in the present study there is a restricted number of GFP-positive cells in each retina, so that it is extremely rare that two labelled cells are side-by-side in the same section. This allowed us to examine the stratification of dendrites from individual cells without the confounding problem of identifying dendrites from overlapping adjacent RGCs; the very rare adjacent cells were not analyzed. All RGCs analyzed are therefore isolated.

The interanimal variability of analyzed cells in our groups (see Tables 1 and 2 and Fig. 3) is comparable to that provided by Tian and Copenhagen [7], who also report different sample size for different groups of animals in their YFP expressing transgenic mouse. Averaging the number of RGCs analyzed in all our groups, an average of 32±10 analyzed cells per retina is found as compared to an average of 43±21 in the Tian and Copenhagen paper.

The number of analyzed cells was independent on our housing and experimental conditions. Indeed, at no age differences were found between the mean number of analyzed cells in non-EE and EE animals [(at P10 t-test, p = 0,482; at P16 t-test p = 0,805; at P30 t-test, p = 0,250); no differences between P30 non-EE, P30 DR and P30 EE-DR (One Way ANOVA, p = 0,407); no differences at P16 in the oligo-treated retinas (One Way ANOVA, p = 0,843)].

We have done the assessment of dendritic stratification patterns by confocal microscope with two methods, on retinal vertical sections and in whole-mount retinas through the entire thickness of the GCL and IPL.

In both cases, retinas were immunostained for ChAT.

For the analysis of RGC dendritic segregation in vertical retinal sections was used a total of 50 mice [non-EE mice at P10 (N = 4 retinas), P16 (N = 4 retinas) and P30 (N = 5 retinas); EE mice at P10 (N = 5 retinas), P16 (N = 5 retinas) and P30 (N = 3 retinas); DR (N = 5 retinas) and EE-DR (N = 4 retinas) mice at P30; P16 EE mice treated with BDNF sense (N = 5 retinas) or antisense oligos (N = 5 retinas) or untreated mice (N = 5 retinas)]. For whole-mount P16 retinas, we analyzed RGC dendritic stratification in 3 non-EE and 3 EE retinas. The number of cells analyzed is given in Fig. 3B and in TABLE 1 and 2.

*Vertical sections* Images were collected using an Olympus Optical confocal microscope with an UPlanApo 20× objective (N.A. = 0,7). Settings for laser intensity, gain, offset and pinhole size were optimised initially and held constant through each experimental session. For each animal, the entire serial order of vertical sections of the retina was acquired, and for each section, confocal series of 1 µm step size were obtained throughout the whole section thickness (25 µm); these confocal series were then averaged and visualized on a single focal plane by Fluoview software.

All images of the GFP RGC dendritic patterning were examined visually at the end of each acquisition and each acquired RGC was assigned to the bistratified or monostratified class according to its pattern of stratification in different sublaminae of the IPL, a protocol similar to that described in Bodnarenko and Chalupa [3], [5], [14] and Deplano et al. [47]. In particular this method is similar to that described in Bodnarenko and Chalupa [3] and the only difference is that we have used ChAT immunostaining to obtain two labelled bands corresponding to the projections of cholinergic amacrine cells. Since these projections run along two discrete sublaminae inside the IPL, called sublamina a (which is made by the two upper IPL strata, below the amacrine cell bodies) and b (made by the other three IPL strata) [1], [2], [10], [11], [6], this immunostaining clearly defines the two reference planes we have used to detect RGCs as bistratified or monostratified. Moreover, ChAT bands are useful to align the different sections on different confocal planes ensuring the dendrites are reconstructed properly. In the cases in which the ChAT bands were not clearly detectable as in the retinal periphery, the RGCs were not analysed.

Since many RGCs have dendritic fields larger than 25 µm, in order to analyze the total extension of dendritic arborizations we have followed their dendritic field on several adjacent vertical serial sections. For each cell all the serial vertical sections containing GFP labeled arborizations were analyzed. We classified one RGC as monostratified if no GFP positive dendritic processes were present on both ChAT planes, as determined from all serial vertical sections examined; as bistratified if GFP positive dendritic arborizations ramified in correspondence of both ChAT planes.

All acquisitions and analysis were performed blind to the age and rearing condition of the animals.

*Whole-mount retinas* We have analyzed RGC segregation at postnatal age 16, age at which the difference in the RGC stratification pattern is particularly evident in EE in comparison to non-EE mice. Images of RGCs from whole-mount retinas were collected using a Leica confocal microscope (CTR6000) with a 40× oil objective (N.A. = 1,25; image size 375 µm×375 µm). Settings for laser intensity, gain, offset and pinhole size were optimised initially and held constant through each experimental session. To quantify the extent of the dendrites of RGCs, z-stack confocal images were taken at 1 µm intervals through the entire thickness of the GCL and IPL. For each analyzed RGC, we have reconstructed both the maximum projection of the acquired stack of images and its 90-degree rotation respect to z-axis by using Leica confocal software. We classified one RGC as monostratified or bistratified using the two ChAT bands as reference planes. The number of RGCs analyzed with this procedure is reported in Table 1 in the shaded rectangles.

Both methods to assess the dendritic stratification gave equivalent results and because the vertical section confocal analysis was less labour intensive, it was used in all our experimental treatments.

### Analysis of BDNF expression in the retina

Images of retinal sections were acquired at 20× magnification using an Olympus Optical confocal microscope with an UPlanApo 20× objective (N.A. = 0,7). To compare different specimens, the parameters of acquisition were optimized at the beginning and then held constant throughout image acquisition. Then, the collected images of the retina were imported to the image analysis system MetaMorph and used to evaluate pixel intensity of cellular immunofluorescence. All image analyses were done blind. The profile of cells into RGC layer was outlined and pixel intensity was measured within this area.

BDNF immunoreactivity levels were calculated as the ratio between the pixel intensity of RGC profiles and the background level, measured in the outer nuclear layer (ONL). Values obtained from at least 5-6 retinal fields were used to calculate the average pixel intensity value per animal.
